# Exploring the Neuropharmacological Potential of *Ligustrum sinense* Relevant to Parkinson's Disease: In Vivo and In Silico Study

**DOI:** 10.1002/cbdv.71799

**Published:** 2026-09-30

**Authors:** Sowmitro Das, S. M. Istiaque Hamim, Md Nazmul Hasan Zilani, Kazi Sneha Chhando, Puza Das, Md Ridoy Hossain, Md. Imtiaz, Jebin Tasmin, Roney Miah, Md. Nazmul Hasan

**Affiliations:** ^1^ Laboratory of Pharmaceutical Biotechnology & Bioinformatics Department of Genetic Engineering and Biotechnology Jashore University of Science and Technology Jashore Bangladesh; ^2^ Faculty of Industrial Sciences and Technology Universiti Malaysia Pahang Al‐Sultan Abdullah Kuantan Pahang Malaysia; ^3^ Department of Pharmacy Jashore University of Science and Technology Jashore Bangladesh; ^4^ Department of Zoology Government Brajalal College Khulna Bangladesh; ^5^ Faculty of Veterinary Science Bangladesh Agricultural University Mymensingh Bangladesh

**Keywords:** antioxidant activity, in silico, in vivo, MAO‐A and MAO‐B, neuropharmacological activity, Parkinson's disease, *Ligustrum sinense*

## Abstract

Medicinal plants are valuable sources of bioactive compounds with potential applications in neurodegenerative disorders. This study investigated the antioxidant, neuropharmacological, and monoamine oxidase (MAO) inhibitory activities of *Ligustrum sinense* (*L. sinense*) leaf extract through in vitro, in vivo, and in silico approaches. Antioxidant activity assessed by the DPPH assay demonstrated moderate free radical scavenging capacity (IC_50_ = 73.56 ± 2.18 µg/mL) compared with l‐ascorbic acid (IC_50_ = 7.29 ± 2.86 µg/mL). Behavioral evaluations using the open field, hole cross, elevated plus maze, and hole board tests revealed no significant sedative effects at 200 and 400 mg/kg, while dose‐dependent anxiolytic‐like activity was observed through increased exploratory and head‐dipping behaviors. Computational analyses, including molecular docking, ADMET profiling, PASS prediction, and molecular dynamics (MD) simulations, identified several phytocompounds with notable affinity toward MAO‐A and MAO‐B, key therapeutic targets in Parkinson's disease (PD). CID‐91697556 exhibited the strongest interaction with MAO‐B (−10.478 kcal/mol), whereas CID‐519540 showed dual inhibitory potential against MAO‐A (−6.901 kcal/mol) and MAO‐B (−8.978 kcal/mol). These compounds demonstrated favorable pharmacokinetic properties and stable protein–ligand interactions. Collectively, *L. sinense* shows promising neuropharmacological potential and may represent a natural source for future PD‐related drug discovery, warranting further experimental validation and bioactive compound isolation.

## Graphical abstract

*Ligustrum sinense* (*L. sinense*) leaf extract exhibited moderate antioxidant activity (DPPH IC_50_ = 73.56 ± 2.18 µg/mL) and was evaluated for neuropharmacological effects at 200 and 400 mg/kg using elevated plus maze, open‐field, hole‐cross, and hole‐board tests. Twenty GC–MS‐derived phytocompounds were screened against monoamine oxidase‐A (MAO‐A) and monoamine oxidase‐B (MAO‐B) through molecular docking, ADMET profi, PASS prediction, and molecular dynamics (MD) simulation analyses. CID‐91697556 showed the strongest MAO‐B binding (−10.478 kcal/mol), while CID‐519540 demonstrated dual MAO‐A/MAO‐B affinity (−6.901/‐8.978 kcal/mol), identifying potential lead compounds for Parkinson's disease (PD) drug discovery.

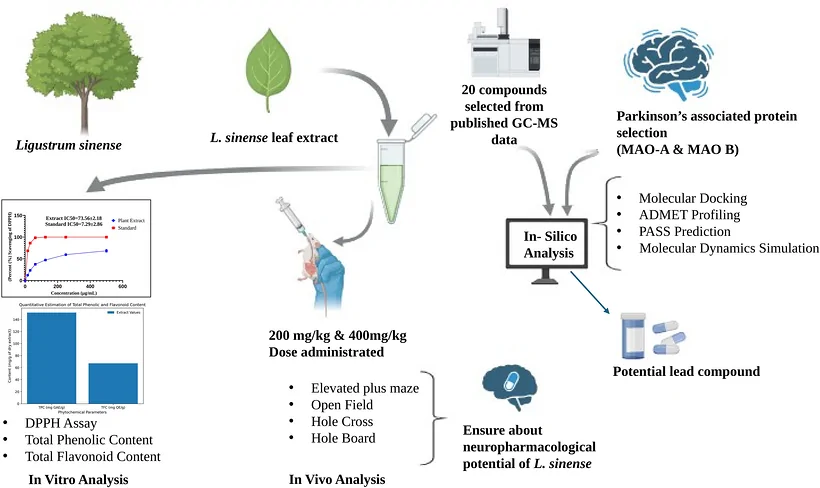

## 1 Introduction

Neurological disorders encompass a diverse range of conditions that impair the central and peripheral nervous systems, involving structures such as the brain, spinal cord, cranial nerves, and neuromuscular junctions. Major examples include Parkinson's disease (PD), epilepsy, brain tumors, multiple sclerosis, Alzheimer's disease (AD), cerebrovascular disorders, neuroinfectious diseases, and neurological complications associated with nutritional deficiencies [1]. These conditions impose a considerable global health burden because of their chronicity, progressive disability, and detrimental effects on quality of life, often necessitating long‐term treatment and disease management. PD is the second most common neurodegenerative disorder worldwide and primarily affects older adults, with its incidence expected to increase alongside population aging and longer life expectancy [2]. Its clinical manifestations include motor symptoms such as resting tremor, rigidity, bradykinesia, and postural instability, together with non‐motor features such as cognitive and mood disturbances that can significantly impair daily functioning [3]. Current therapeutic strategies for PD primarily focus on alleviating symptoms rather than halting or reversing the underlying neurodegenerative process. In addition, long‐term treatment may be associated with reduced therapeutic efficacy and treatment‐related adverse effects [4]. Therefore, the development of safer and more effective interventions that target the mechanisms driving neurodegeneration and may potentially slow disease progression remain an important unmet need.

A defining pathological characteristic of PD is the gradual loss of dopaminergic neurons within the substantia nigra pars compacta [5]. Although the underlying causes of this neurodegenerative process have not been fully elucidated, several interconnected mechanisms are believed to contribute to disease development. Among these, neuroinflammation plays a significant role and is associated with increased production of pro‐inflammatory cytokines, activation of glial cells, and enhanced expression of inflammation‐related proteins, collectively promoting neuronal injury and degeneration [6, 7].

Oxidative imbalance is considered a key pathological factor in PD, with substantial evidence indicating its involvement in dopaminergic neuronal degeneration. Postmortem analyses of the substantia nigra in PD patients have revealed elevated lipid peroxidation, including 4‐hydroxyl‐2‐nonenal (HNE), increased protein carbonylation, and oxidative damage to nucleic acids, reflected by biomarkers such as 8‐hydroxy‐deoxyguanosine and 8‐hydroxyguanosine. Similarly, neurotoxin‐based experimental models involving 6‐hydroxydopamine (6‐OHDA), rotenone, and MPTP have demonstrated progressive loss of dopaminergic neurons associated with excessive reactive oxygen species (ROS) generation [8, 9]. Genetic susceptibility also contributes substantially to PD pathogenesis, with mutations in SNCA, PARK2, DJ‐1, PINK1, LRRK2, and ATP13A2 linked to familial forms of the disease. These genes regulate critical cellular functions, including mitochondrial homeostasis, protein aggregation, and responses to cellular stress, highlighting shared molecular pathways in neurodegeneration [10, 11, 12]. Because conventional PD treatments may cause adverse effects and lose effectiveness during prolonged use, increasing attention has been directed toward natural‐product‐based interventions. Medicinal plants provide diverse bioactive compounds, with *Mucuna pruriens* and *Withania somnifera* exhibiting promising neuroprotective potential against PD‐related pathology [13, 14].

Monoamine oxidases (MAOs) are mitochondrial flavoprotein enzymes that catalyze the oxidative deamination of monoamine neurotransmitters, including dopamine, serotonin, norepinephrine, and melatonin [15]. Monoamine oxidase (MAO) occurs in two isoforms, monoamine oxidase‐A (MAO‐A) and monoamine oxidase‐B (MAO‐B), which exhibit distinct substrate selectivity and tissue localization. MAO‐A primarily oxidizes serotonin, norepinephrine, and melatonin, while MAO‐B preferentially metabolizes phenylethylamine and benzylamine [16]. In the brain, MAO‐A is predominantly associated with dopaminergic neurons, while MAO‐B is mainly expressed in astrocytes and serotonergic neurons [17, 18]. Genetic variations in the MAO‐B gene have also been linked to increased susceptibility to PD [19]. Furthermore, MAO‐B converts the pro‐neurotoxin MPTP into MPP^+^, a toxic metabolite that selectively damages dopaminergic neurons, making MAO‐B an important contributor to PD pathogenesis and a well‐established therapeutic target [20].

*Ligustrum sinense* (*L. sinense*), a plant native to China, Vietnam, and Laos, has traditionally been used in Chinese medicine for its purported benefits in liver and kidney nourishment and vision improvement [21]. Phytochemical investigations of *Ligustrum* *species* have identified diverse bioactive constituents, including flavonoids, terpenoids, iridoids, and phenylethanoid glycosides, which exhibit notable antioxidant and anti‐inflammatory activities [21, 22, 23]. Antioxidant glycosides have also been isolated from *L. sinense* leaves, further supporting its pharmacological potential [22]. Although botanicals such as *Mucuna pruriens* and *Withania somnifera* have demonstrated established neuroprotective effects in PD [13, 14], the neuropharmacological potential of *L. sinense* had not been investigated in a PD‐relevant context before the present study. Its rich antioxidant and anti‐inflammatory phytochemical composition therefore represent a promising and previously unexplored avenue for natural‐product‐based therapeutic discovery. Notably, *L. sinense* leaves are also used in the preparation of Ku‐Ding‐Cha, a bitter herbal tea traditionally consumed in China for its purported anti‐inflammatory and metabolic health benefits, providing additional support for its traditional use and suitability for pharmacological investigation [24].

However, comprehensive studies investigating the neuropharmacological effects and underlying molecular mechanisms of *L. sinense* in the context of PD remain limited [22]. Accordingly, this study aimed to evaluate the neuropharmacological potential of *L. sinense* through a range of in vivo behavioral assays, supported by complementary in silico investigations. The computational analyses examined the possible interactions between its phytoconstituents and MAO‐A and MAO‐B. As key regulators of monoamine metabolism, these enzymes are also linked to oxidative stress and neuronal dysfunction associated with PD. By combining experimental observations with computational predictions, this study aims to provide preliminary insights into the sedative and anxiolytic activities of *L. sinense* and to examine whether its bioactive constituents exhibit interaction profiles consistent with MAO inhibition. The findings are intended to contribute to the existing knowledge base and support future studies focused on mechanistic validation and therapeutic relevance.

## 2 Materials and Methods

### 2.1 Chemicals and Reagents

All chemicals and reagents utilized in this study were of analytical grade and used as received without any additional purification. Gallic acid (GA), sodium carbonate, quercetin, aluminum chloride, 2,2‐diphenyl‐1‐picrylhydrazyl (DPPH), ascorbic acid (AA), and ethanol were purchased from Merck (Darmstadt, USA). Diazepam, which served as the reference standard for the neuropharmacological assays, was obtained from Square Pharmaceuticals PLC, Bangladesh. All remaining solvents and reagents used during the experimental procedures were of standard laboratory grade and sourced from reliable commercial suppliers.

### 2.2 Plant Material Collection and Extract Preparation

Fresh *L. sinense* leaves were collected from Jashore, Bangladesh, authenticated by the Bangladesh National Herbarium, and deposited under voucher No. DACB‐51459. The leaves were washed, shade‐dried for 10 days, powdered, and extracted by maceration of 100 g of powder in 500 mL of absolute ethanol for 10 days at room temperature. The extract was filtered, concentrated under reduced pressure at 50°C using a rotary evaporator, yielding 4.94% (w/w) crude extract, and stored at 4°C until further analysis.

### 2.3 Total Phenol Content (TPC)

The total phenolic content (TPC) of the extract was assessed using the Folin–Ciocalteu colorimetric assay, with minor modifications to a previously reported method [25]. The TPC was determined using the Folin–Ciocalteu colorimetric method, in which phenolic compounds produce a blue‐colored complex with the reagent. Briefly, 1 mL of extract (500 µg/mL) was mixed with 5 mL of 10% (v/v) Folin–Ciocalteu reagent and left for 5 min. Then, 4 mL of 7.5% (w/v) sodium carbonate was added, followed by gentle vortexing for 15 s. The mixture was incubated at 40°C for 30 min, and absorbance was recorded at 765 nm using a UV–visible spectrophotometer. TPC was quantified against a GA calibration curve and expressed as mg GA equivalents per gram of dry extract (mg GAE/g).

The total TPC was calculated using the following equation:

$$\mathrm{TPC},\;X=\frac{C\times V}{M}$$

where *X* represents the total TPC, *C* is the concentration of GA equivalent obtained from the absorbance, *V* is the volume of the extract solution (mL), and *M* is the mass of the extract (g).

### 2.4 Total Flavonoid Content (TFC)

The total flavonoid content (TFC) of the plant extract was quantified using the aluminum chloride colorimetric method, with minor modifications to a previously reported procedure [26]. The extract was prepared in methanol at 100 mg/mL, while quercetin standards were serially diluted in methanol over a concentration range of 0–100 mg/mL. For the assay, 2.0 mL of the sample or standard was mixed with 0.1 mL of 10% (w/v) aluminum chloride and 0.1 mL of 1 M potassium acetate. After gentle vortexing, the mixture was incubated at room temperature for 5 min to facilitate complex formation. Absorbance was measured at 415 nm using a UV–visible spectrophotometer (Perkin–Elmer Lambda 950, UK). TFC was calculated using the quercetin calibration curve and expressed as mg quercetin equivalents per gram of dry extract (mg QE/g DE).

### 2.5 2,2‐Diphenyl‐1‐picrylhydrazyl (DPPH) Free Radical Scavenging Assay

The DPPH radical‐scavenging activity of the ethanolic plant extract was assessed using a slightly modified previously reported method [27]. Ethanolic extract solutions (15.625–500 µg/mL) were evaluated for DPPH radical‐scavenging activity using AA as the standard antioxidant. Briefly, 1 mL of each sample or standard was mixed with 6 mL of freshly prepared 0.004% (w/v) DPPH in ethanol, vortexed, and incubated in the dark at room temperature for 30 min. Absorbance was recorded at 517 nm using a UV–visible spectrophotometer (Perkin–Elmer Lambda 950, UK), with DPPH solution alone used as the control. The radical‐scavenging activity was calculated as follows:

$$\mathrm{Scavenging}\;\mathrm{activity}\left(\%\right)=\left[1-\left(\frac{A_{\mathrm{sample}}}{A_{control}}\right)\right]\times 100$$

where $A_{\mathrm{Sample}\;}$ and $A_{\mathrm{Control}}$ denote the absorbances of the sample and control, respectively. The IC_50_ value, defined as the concentration of the extract necessary to neutralize 50% of the DPPH radicals, was calculated from the corresponding dose–response curve and compared with the IC_50_ value obtained for AA.

### 2.6 In Vivo Studies

#### 2.6.1 Experimental Animals

Healthy male Swiss albino mice (6‐weeks‐old; 20–28 g) were used for the in vivo study. The animals were maintained at 25.0°C ± 1.0°C, 50%–70% relative humidity, and a 12 h light/dark cycle, with standard laboratory feed and water available ad libitum. The mice were obtained from the Pharmacy Research Laboratory and Animal House, Jashore University of Science and Technology, Bangladesh.

Prior to the experiments, the animals were acclimatized to laboratory conditions to minimize stress and experimental variation. All procedures complied with the European Community Guidelines for the Care and Use of Laboratory Animals (EEC Directive, 1986; 88/609/EEC). Ethical clearance was obtained from the Ethical Review Committee of the Faculty of Biological Science and Technology, Jashore University of Science and Technology (Ref: ERC/FBS/JUST/2022‐114).

#### 2.6.2 Dosing Groups

For each behavioral assessment, the experimental mice were randomly distributed into groups, with 20 animals used for each test: open field test (OFT), hole cross test (HCT), elevated plus maze (EPM), and hole board test (HBT). Each behavioral assay consisted of four groups, containing five mice per group (*n* = 5), giving a total of 80 animals for all experiments [28, 29]. The plant extract was administered orally (p.o.) at doses of 200 and 400 mg/kg body weight, whereas the control animals received distilled water orally at 10 mL/kg body weight. Diazepam served as the standard reference drug and was administered intraperitoneally (i.p.) at the doses specified for each respective behavioral experiment.

### 2.7 Neuropharmacological Activities

#### 2.7.1 Sedative Activity

##### 2.7.1.1 Open Field Test (OFT)

The OFT was conducted to assess spontaneous locomotor and exploratory behavior according to the procedure reported by [29], with minor modifications. The test apparatus was a square Plexiglas arena measuring 50 × 50 × 40 cm (height), with the floor divided into marked squares using black lines to enable movement assessment. Diazepam was administered intraperitoneally at 1 mg/kg [29], whereas the remaining treatments were given orally (p.o.) before the test. Each mouse was placed individually at the center of the arena, and locomotor activity was assessed by counting the squares crossed with all four paws during a 3‐min observation period. Measurements were performed at 0, 30‐, 60‐, 90‐, and 120‐min following treatment. After each trial, the arena was cleaned with 70% ethanol and completely dried to prevent odor‐related behavioral bias.

##### 2.7.1.2 Hole‐Cross Test (HCT)

The HCT was conducted to assess exploratory and locomotor behavior using a previously established procedure [30]. The apparatus comprised a rectangular wooden cage measuring 30 × 20 × 14 cm, which was separated into two equal compartments by a fixed partition. A circular aperture of approximately 3 cm in diameter was positioned at the center of the partition to permit movement between the compartments. Diazepam (1 mg/kg, i.p.) was used as the standard reference drug [30], whereas the other treatments were administered orally (p.o.) prior to testing. At 0, 30‐, 60‐, 90‐, and 120‐min following treatment, each mouse was individually placed in the apparatus, and spontaneous locomotor activity was evaluated by recording the number of times it crossed through the aperture between the two compartments during a 3‐min observation period. The apparatus was cleaned with 70% ethanol and thoroughly dried between trials to minimize residual olfactory cues.

#### 2.7.2 Anxiolytic Activity

##### 2.7.2.1 Hole‐Board Test (HBT)

HBT was employed to assess exploratory activity and anxiety‐related behavior in mice. The experimental apparatus consisted of a wooden platform measuring 40 × 40 cm, containing 16 uniformly distributed holes, each approximately 3 cm in diameter and 2.2 cm in depth. The board was elevated above the floor to allow assessment of exploratory responses during the test [31].

Behavioral assessment was carried out 60 min after oral administration of the test extracts and the control. The standard anxiolytic, diazepam (0.5 mg/kg, i.p.), was administered 30 min before testing due to its rapid onset of action after intraperitoneal administration. This specific dose of diazepam has been validated to produce significant anxiolytic effects without inducing sedation or impairing locomotor activity in rodents [32, 33]. Each mouse was individually positioned at the center of the elevated board and permitted to explore the apparatus for 5 min. Exploratory activity was assessed by counting the number of head‐dipping responses, defined as the insertion of the animal's head into a hole to approximately eye level. After each trial, the apparatus was thoroughly cleaned with 70% ethanol and allowed to dry to minimize the influence of residual olfactory cues on subsequent behavioral observations.

##### 2.7.2.2 Elevated Plus‐Maze Tests (EPM)

The EPM test was employed to evaluate anxiety‐like behavior in mice according to a previously established protocol [34]. The EPM comprised two open arms (35 × 5 cm) and two enclosed arms (30 × 5 × 15 cm) fitted with 15‐cm‐high opaque walls, all connected to a central platform measuring 5 × 5 cm. The maze was positioned 60 cm above the floor.

Individual mice were positioned on an open arm with their heads oriented toward the central platform and allowed to move freely throughout the maze for 5 min. An arm entry was considered valid when the mouse's four paws had completely crossed into the arm. The time spent and frequency of entries in both open and closed arms were recorded. Open‐arm time was expressed as (open‐arm time/total test duration) × 100, whereas open‐arm entry percentage was calculated as (number of open‐arm entries/total entries) × 100. These measures served as the principal indicators of anxiety‐like behavior. The plant extracts and control treatment were administered orally 60 min before testing, whereas diazepam (0.5 mg/kg, i.p.) was given 30 min before the EPM assessment as the reference anxiolytic [34]. Following each trial, the apparatus was cleaned thoroughly with 70% ethanol and dried to minimize residual odor cues that could influence subsequent behavioral responses.

### 2.8 In Silico Study

#### 2.8.1 Ligands Selection and Library Preparation

A total of 22 phytocompounds were selected for in silico analysis based on our previously reported GC–MS profiling of *L. sinense* (Table S1) [35]. Of these, 20 compounds were further selected for molecular docking based on their availability and suitability for downstream in silico analyses. The 3D structures of the selected phytocompounds and control drug were retrieved in SDF (Structure Data File) format from the PubChem database. The compounds were subsequently prepared for in silico analysis through geometry optimization and energy minimization prior to molecular docking.

### 2.9 Molecular Docking

#### 2.9.1 Ligands Preparation

The 20 selected ligands were processed for molecular docking using the Ligand Preparation workflow in Maestro (Schrödinger Release 2020‐3, Schrödinger, LLC, New York, NY, USA). Epik was employed to generate the appropriate ionization states within a target pH range of 7.0 ± 2.0, followed by geometric optimization using the OPLS3e force field [36]. For each ligand, up to 32 stereoisomeric and tautomeric conformations were generated, applying an RMSD cutoff of 1.0 Å. This approach enabled the retention of structurally unique ligand conformations while minimizing the inclusion of redundant molecular states.

#### 2.9.2 Protein Selection and Preparation

The 3D tertiary structures of MAO‐A and MAO‐B were retrieved from the RCSB Protein Data Bank (https://www.rcsb.org/) with the PDB IDs: 2Z5X and 4A79, respectively [37, 38]. The obtained structures were prepared using the Protein Preparation Wizard implemented in Maestro (Schrödinger Release 2020‐3).

Protein preprocessing involved assignment of bond orders, addition of hydrogen atoms, and correction of formal charges. Disulfide bonds were identified and assigned, while missing side chains and loops were reconstructed using the Prime module. Metal coordination states were treated by assigning zero‐order bonds where appropriate. Epik was used to assign protonation states at pH 7.0 ± 2.0, followed by hydrogen‐bond optimization with PROPKA at pH 7.0. Water molecules beyond 5 Å from the binding site were removed. The protein structure was then minimized using the OPLS3e force field with heavy‐atom restraints of 0.30 Å to resolve steric clashes while preserving its overall conformation.

#### 2.9.3 Binding Site and Grid Box Generation

The binding sites were defined using the co‐crystallized ligands, and receptor grids were generated in Maestro (Schrödinger Release 2020‐3) by centering the grid boxes on the corresponding ligand‐binding pockets [39]. For MAO‐A, the grid center coordinates were set at *X* = 38.2129, *Y* = 26.5090, and *Z* = –13.3977, with grid dimensions of *X*: 23.7690 Å, *Y*: 20.4392 Å, and *Z*: 24.6578 Å. Similarly, for MAO‐B, the grid center was defined at *X* = 51.3474, *Y* = 156.1342, and *Z* = 29.7825, with dimensions of *X*: 26.9918 Å, *Y*: 29.8692 Å, and *Z*: 30.3826 Å.

#### 2.9.4 Site‐Specific Molecular Docking

Molecular docking is a computational technique used to predict ligand‐binding poses, affinity, and interactions with target proteins [40]. In this study, site‐specific docking was conducted using the extra precision (XP) protocol in Maestro (Schrödinger Release 2020‐3, Schrödinger, LLC, New York, NY, USA). The resulting protein–ligand complexes were exported in PDB format and analyzed using BIOVIA Discovery Studio 2025 to characterize hydrogen bonds, hydrophobic contacts, non‐bonded interactions, and other binding patterns associated with complex stability and potential biological activity [41]. Furthermore, the docking protocol was validated by redocking the co‐crystallized ligands into the active sites of MAO‐A (PDB ID: 2Z5X) and MAO‐B (PDB ID: 4A79).

#### 2.9.5 ADMET Profiling

The ADMET (Absorption, Distribution, Metabolism, Excretion, and Toxicity) properties of the selected phytochemicals, along with the reference drug, were systematically evaluated using the SwissADME (http://www.swissadme.ch/) and pkCSM (http://biosig.unimelb.edu.au/pkcsm/prediction) web‐based prediction tools [42, 43].

#### 2.9.6 Ligands Activity Analysis via PASS Prediction

The biological activity profiles of the selected phytochemicals were predicted using the PASS online server (Prediction of Activity Spectra for Substances). Each compound was submitted as a SMILES representation, and the corresponding probabilities of activity (Pa) and inactivity (Pi) were estimated according to their molecular structural characteristics. The activities that involved Pa > Pi were chosen, and particular attention was paid to the treatment of antiparkinsonian, antineurotic, neurotrophic factor improvement, neurotransmitter antagonism, and acute neurologic disorders [44].

#### 2.9.7 Molecular Dynamics (MD) Simulation

Molecular dynamics (MD) simulations were performed to investigate the stability, flexibility, and conformational dynamics of the protein–ligand systems under near‐physiological conditions. Simulations were conducted using the Desmond package (Schrödinger Release 2020–3) on a Linux‐based computational platform [45]. Prepared MAO‐A and MAO‐B crystal structures served as the starting coordinates, with system parameters generated using the OPLS3e force field [36]. Each system was solvated using the TIP3P water model [46] in an orthorhombic periodic box with a 10 Å buffer, followed by addition of counterions and 0.15 M NaCl to maintain charge neutrality and physiological ionic strength [45].

Before production, the systems underwent energy minimization and restrained equilibration, including NVT heating to 300 K followed by NPT equilibration at 1 atm [47]. Temperature and pressure were controlled using the Nose–Hoover thermostat and Martyna–Tobias–Klein barostat, respectively [45]. Subsequently, 100 ns unrestrained MD simulations were performed under NPT conditions at 300 K and 1 atm using a 2 fs timestep. Long‐range electrostatics were treated with the particle mesh ewald (PME) method, and trajectories were saved periodically for subsequent structural and interaction analyses.

#### 2.9.8 Statistical Analysis

All experiments were performed in triplicate, with results presented as mean ± SEM. In vivo data were analyzed using one‐ or two‐way ANOVA followed by Dunnett's multiple‐comparison test in GraphPad Prism 10. Statistical significance was set at *p* < 0.05, with *p* < 0.01 and *p* < 0.001 indicating higher significance levels.

## 3 Results

### 3.1 Total Phenolic Content (TPC) and Total Flavonoid Content (TFC)

Based on the standard GA calibration curve (*Y* = 0.00522X ‐ 0.00290; *R* ^2^ = 0.997), the TPC of the extract was determined to be 151.6 mg GAE/g of dry extract. The TFC was measured at 67.25 mg QE/g of dry extract.

### 3.2 2,2‐Diphenyl‐1‐picrylhydrazyl (DPPH) Free Radical Scavenging Assay

Both the plant extract and AA demonstrated concentration‐dependent DPPH radical‐scavenging activity. The extract exhibited an IC_50_ value of 73.56 ± 2.18 µg/mL, whereas AA showed a lower IC_50_ of 7.29 ± 2.86 µg/mL (Figure 1).

**FIGURE 1:**
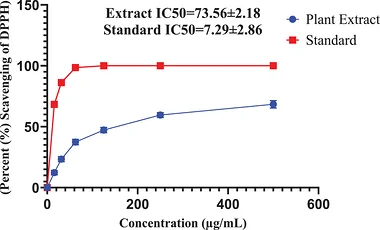
DPPH free radical scavenging assay.

### 3.3 Neuropharmacological Activities of *L. sinense*

#### 3.3.1 Sedative Activity: Open Field Test (OFT) and Hole‐Cross Test (HCT)

The effects of *L. sinense* extract on locomotor and exploration behavior were evaluated using the OFT and HCT (Figure 2a,b). In the OFT, diazepam (1 mg/kg, i.p.) significantly reduced locomotor activity at all time points (30–120 min), with highly significant effects at 30–90 min (***p < 0.001) and a lesser effect at 120 min (*p < 0.05). Similarly, in the HCT, diazepam significantly decreased the number of hole crossings at all observation periods, showing stronger effects at 30–60 min (**p < 0.01) and comparatively lower significance at 90–120 min (*p < 0.05), confirming its sedative activity.

**FIGURE 2:**
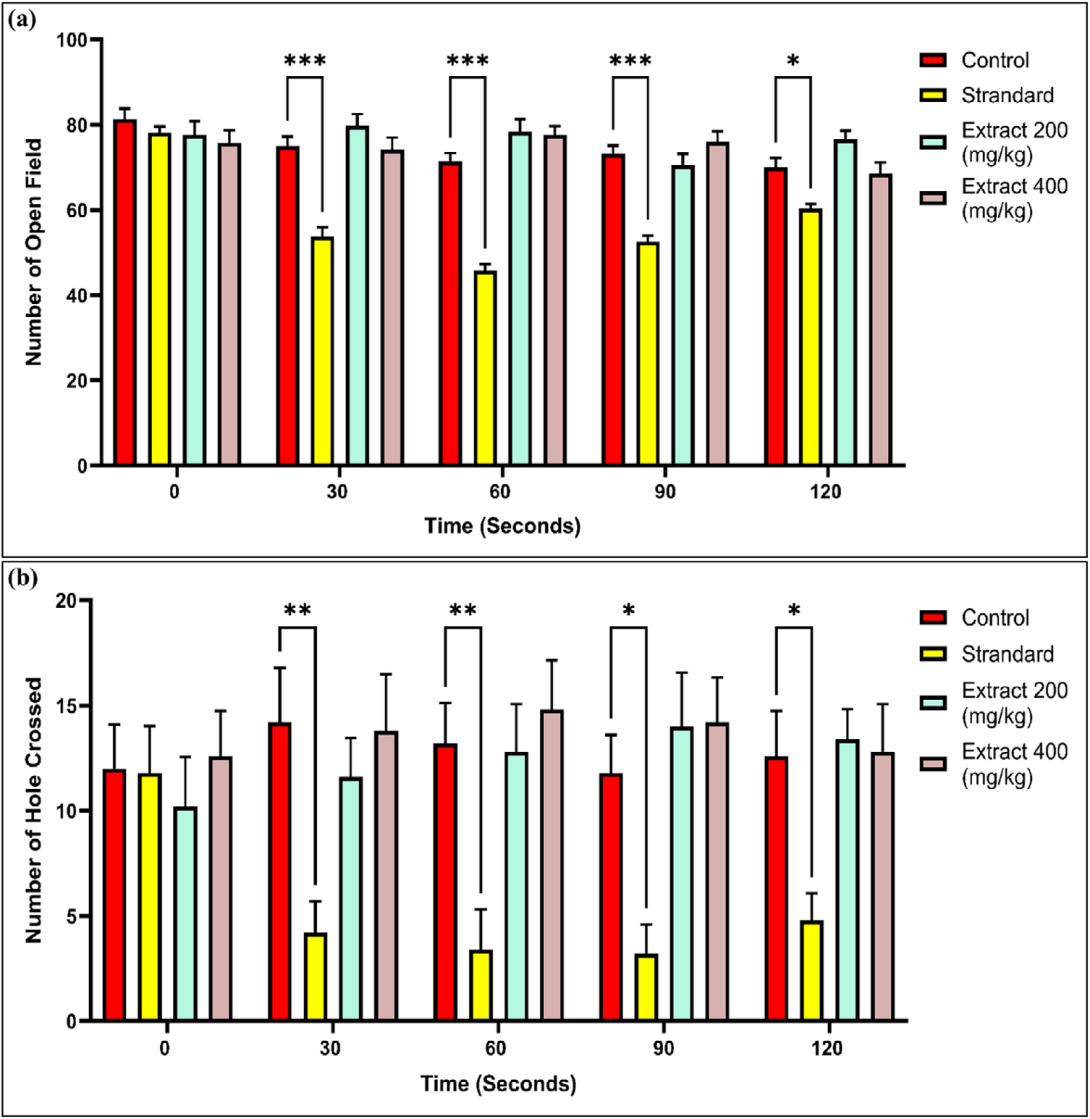
Sedative activity of *L. sinense* in (a) OFT and (b) HCT. Values are mean ± SEM (*n* = 5); *p < 0.05, **p < 0.01, ***p < 0.001 versus control.

In contrast, administration of *L. sinense* extract at 200 and 400 mg/kg did not result in any significant changes in locomotor or exploration activity relative to the control group. A slight, non‐significant increase in activity was observed in the extract‐treated mice, particularly at the later observation periods, suggesting that the extract did not exhibit a sedative effect at the doses tested.

#### 3.3.2 Anxiolytic Activity: Elevated Plus‐Maze Test (EPM) and Hole‐Board Test (HBT)

In the EPM test, diazepam (0.5 mg/kg) significantly increased the percentage of time spent in the open arms to 32.2% ± 2.59% (**p < 0.001) compared with 10.2% ± 1.65% in the control group (Figure 3a). Similarly, *L. sinense* extract demonstrated a dose‐dependent enhancement in open‐arm exploration. Significant increases were observed at 200 mg/kg (19.8% ± 2.65%; *p < 0.05, *p* = 0.031) and 400 mg/kg (24.6% ± 2.54%; *p < 0.05, *p* = 0.002), indicating an anxiolytic‐like effect of the extract.

**FIGURE 3:**
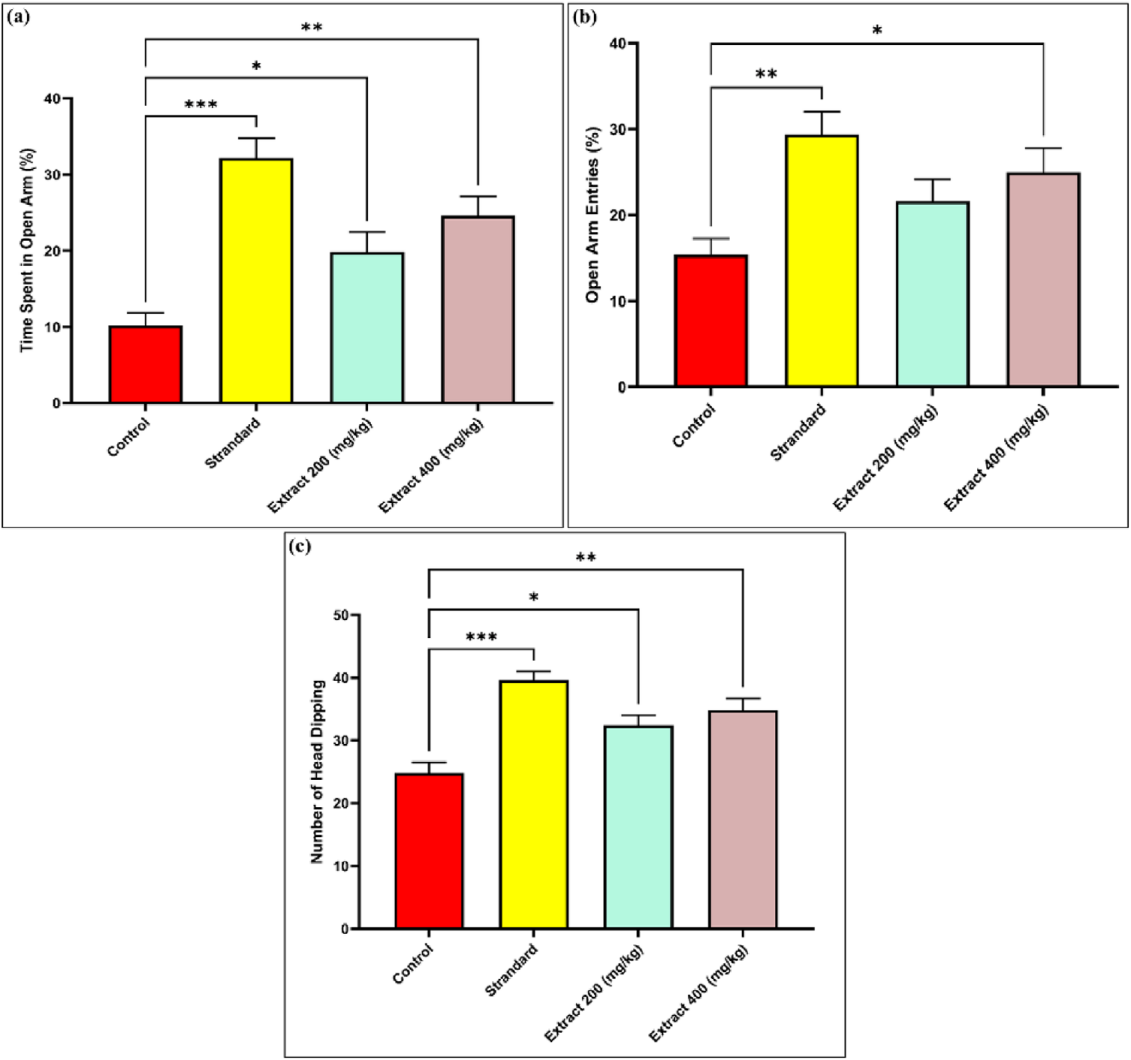
Anxiolytic activity of *L. sinense* extract in mice. (a) Open arm time (%), (b) open arm entries (%), and (c) number of head dips in the hole‐board test. Values are mean ± SEM (*n* = 5). *p < 0.05, **p < 0.01, ***p < 0.001 versus control group.

A similar pattern was observed in the percentage of open‐arm entries (Figure 3b). Diazepam significantly increased open‐arm entries to 26.6% ± 2.80% (**p < 0.01) compared with 15.4% ± 1.86% in the control group. The *L. sinense* extract at 200 mg/kg produced a moderate increase to 21.6% ± 2.54%; however, this change did not reach statistical significance. In contrast, administration of the extract at 400 mg/kg significantly elevated the percentage of open‐arm entries to 25.0% ± 2.77% (*p < 0.05; *p* = 0.037).

In the HBT (Figure 3c), diazepam significantly increased the number of head‐dipping responses to 39.6 ± 1.36 (**p < 0.001) compared with 24.8 ± 1.71 in the control group. Similarly, *L. sinense* extract produced a significant increase in head‐dipping behavior at both 200 mg/kg (32.04 ± 1.63; *p* < 0.05, *p* = 0.014) and 400 mg/kg (34.8 ± 1.90; *p < 0.01, *p* = 0.002), indicating enhanced exploratory activity following extract administration. Collectively, these findings indicate that *L. sinense* extract possesses dose‐dependent anxiolytic‐like activity in both the EPM and HBT.

### 3.4 In Silico Study

#### 3.4.1 Molecular Docking Validation

The molecular docking protocol was validated through redocking of the co‐crystallized ligands into the active sites of MAO‐A (PDB ID: 2Z5X) and MAO‐B (PDB ID: 4A79). The reliability of the docking procedure was assessed by calculating the root‐mean‐square deviation (RMSD) between the experimentally determined crystallographic ligand poses and the corresponding redocked poses. The validation results demonstrated excellent agreement between the crystallographic and predicted binding conformations, with RMSD values of 0.2596 Å for MAO‐A and 0.2169 Å for MAO‐B, respectively (Figure 4a,b). Both RMSD values were well below the commonly accepted threshold of 2.0 Å [40], confirming the high accuracy and reproducibility of the applied docking protocol. These findings indicate that the docking parameters were appropriate for reliably reproducing the experimentally observed ligand‐binding orientations and, therefore, were suitable for subsequent docking analyses of the selected compounds against MAO‐A and MAO‐B.

**FIGURE 4:**
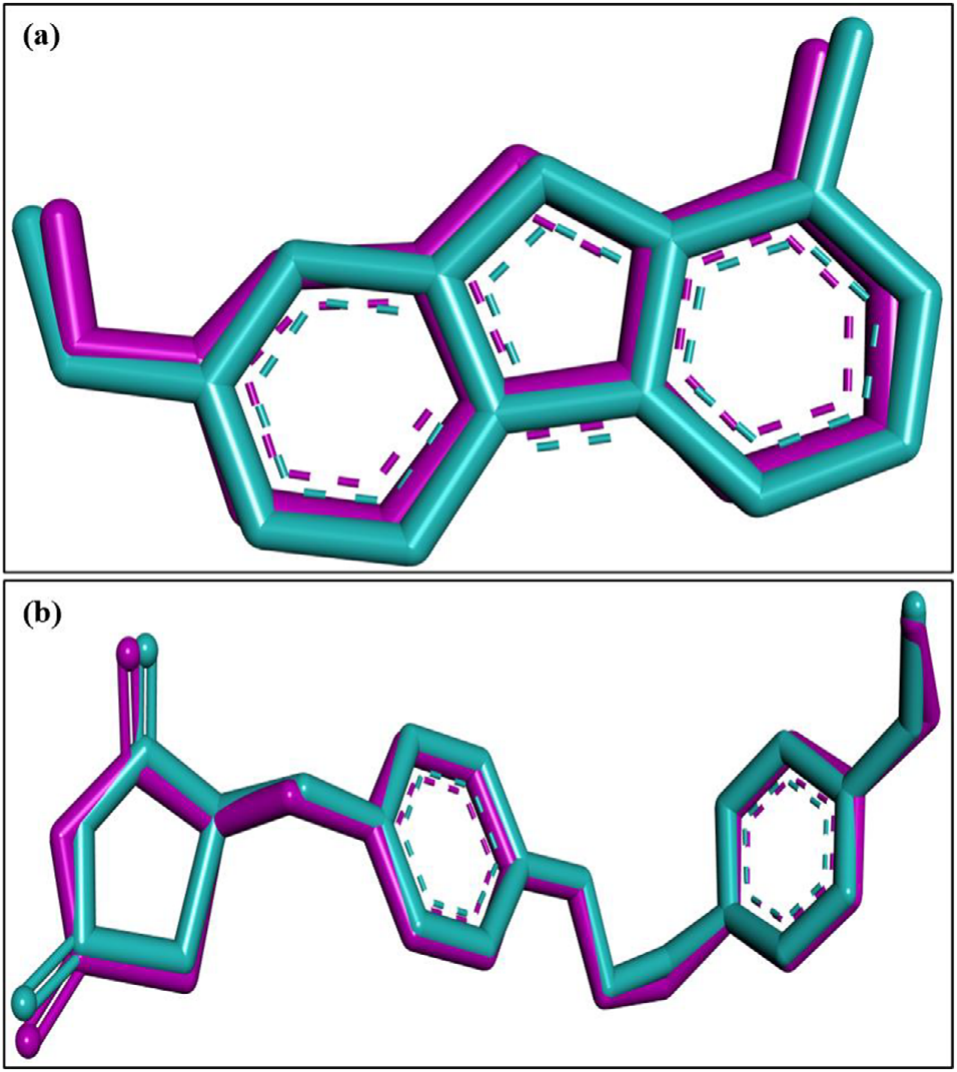
Docking validation of (a) MAO‐A and (b) MAO‐B through redocking of the co‐crystallized ligands, showing RMSD values of 0.2596 and 0.2169 Å, respectively.

#### 3.4.2 Molecular Docking

Molecular docking predicts ligand binding poses and affinities within protein active sites, aiding the identification of potential therapeutic candidates [48]. In this study, docking analysis was performed to investigate the interaction profiles of 20 phytochemical compounds from our previous study [35] against (MAO‐A, PDB ID: 2Z5X) and (MAO‐B, PDB ID: 4A79), which are key enzymatic targets implicated in PD (Table S2). Among the 20 compounds evaluated, those exhibiting the most favorable docking scores and interaction profiles were selected for visualization in Figures 5 and 6, and summarized in Table 1, and chosen for further in silico studies.

**FIGURE 5:**
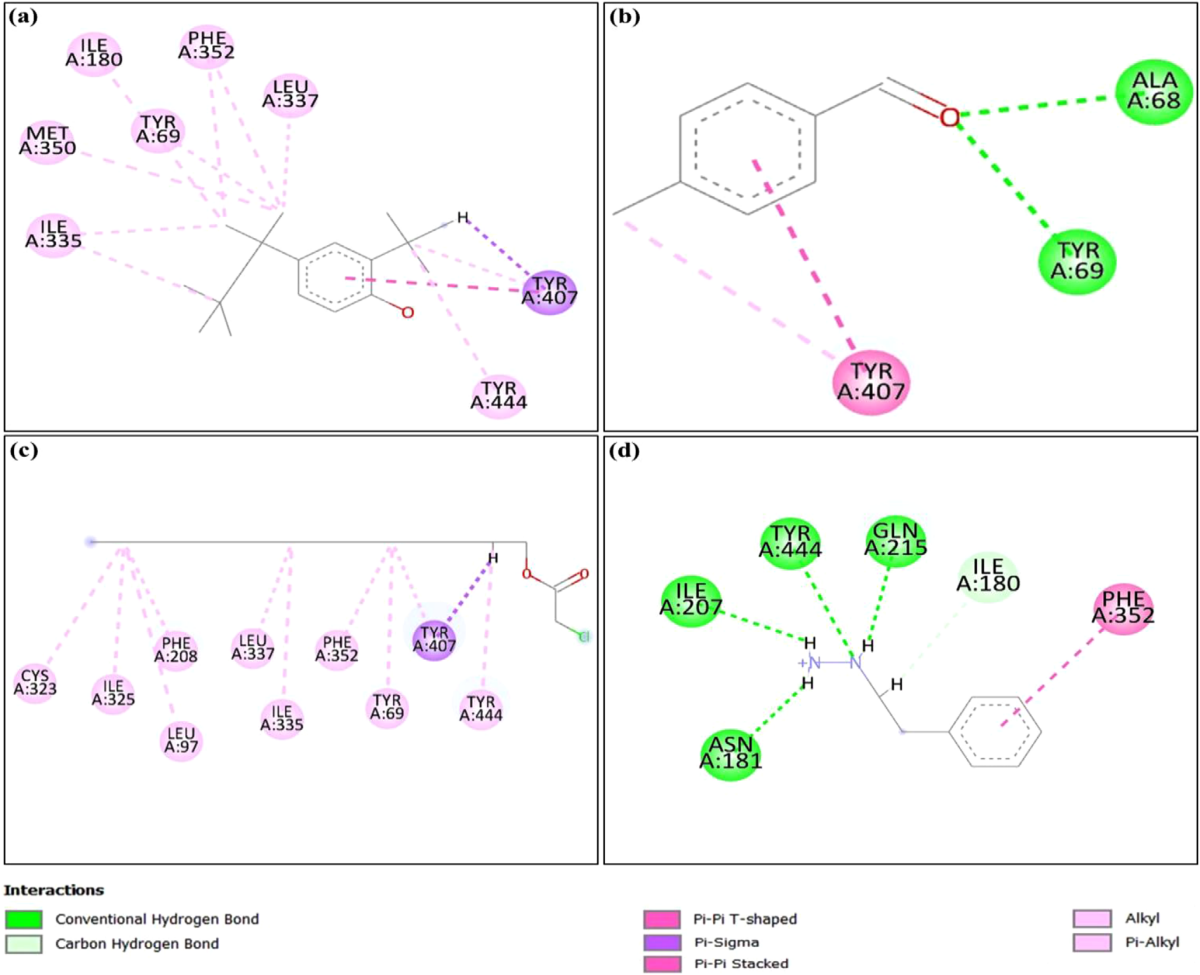
Protein–ligand interaction profiles between MAO‐A protein (PDB ID: 2Z5X) and the selected ligands, including the control inhibitor, obtained from molecular docking analysis. The two‐dimensional interaction diagrams depict the key amino acid residues and bonding interactions involved in complex stabilization between MAO‐A and (a) CID‐605573, (b) CID‐7725, (c) CID‐519540, and (d) CID‐3675.

**FIGURE 6:**
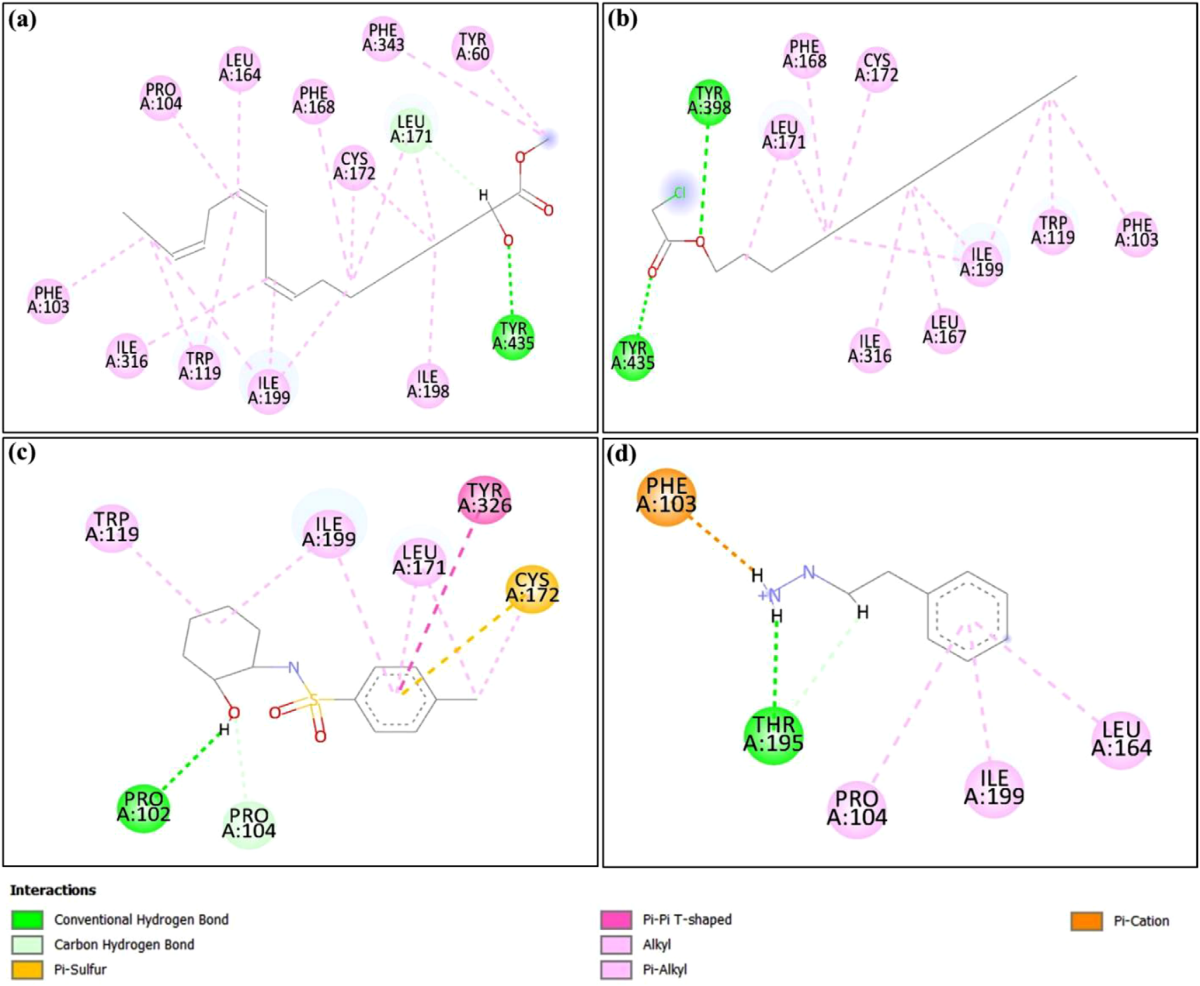
Protein–ligand interaction profiles between MAO‐B protein (PDB ID: 4A79) and the selected ligands, including the control inhibitor, obtained from molecular docking analysis. The two‐dimensional interaction diagrams depict the key amino acid residues and bonding interactions involved in complex stabilization between MAO‐B and (a) CID‐91697556, (b) CID‐519540, (c) CID‐575170, and (d) CID‐3675.

**TABLE 1:** The binding affinities of the top‐ranked ligands toward the target protein, along with their detailed intermolecular interactions.

Ligands for human MAO‐A (PDB ID: 2Z5X)			
		Amino acid interaction	
Compound name (PubChem CID)	Binding score (Kcal/mol)	Hydrogen bond interaction	Hydrophobic bond interaction
Phenol, 2‐(1,1‐dimethyl ethyl)‐4‐(1,1,3,3‐tetra methylbutyl (CID‐605573)	−9.208	**—**	TYR69, ILE180, ILE335, LEU337, MET350, PHE352, TYR407, TYR444
4‐Methylbenzaldehyde (CID‐7725)	−7.41	ALA68, TYR69	TYR407
Tetradecyl chloroacetate (CID‐519540)	−6.901	**—**	TYR69, LEU97, PHE208, CYS323, ILE325, ILE335, LEU337, PHE352, TYR407, TYR444
Phenelzine‐ (control) (CID‐3675)	−6.196	ILE180, ASN181, ILE207, GLN215, TYR444	PHE352

**Ligands for Human MAO‐BB (PDB ID: 4A79)**			
Methyl 2‐hydroxy‐octadeca‐9,12,15‐trienoate (CID‐91697556)	−10.478	LEU171, TYR435	TYR60, PHE103, PRO104, TRP119, LEU164, PHE168, CYS172, ILE198, ILE199, ILE316, PHE343
Tetradecyl chloroacetate (CID‐519540)	−8.978	TYR398, TYR435	PHE103, TRP119, LEU167, PHE168, LEU171, CYS172, ILE199, ILE316
1‐Benzenesulfonamide, N1‐(2‐hydroxycyclohex yl)‐4‐methyl (CID‐575170)	−8.962	PRO102, PRO104	TRP119, LEU171, CYS172, ILE199, TYR326
Phenelzine‐(Control) (CID‐3675)	−6.437	THR195	PHE103, PRO104, LEU164, ILE199

The docking analysis revealed that several selected compounds exhibited strong binding affinities toward MAO‐A and MAO‐B, with some showing more favorable predicted binding scores than the reference inhibitor, phenelzine (Table 1). Phenelzine was selected as the reference inhibitor based on its established activity against both MAO isoforms. Clinically, phenelzine is an irreversible, non‐selective MAO inhibitor that inhibits both MAO‐A and MAO‐B and has been reported to achieve response rates exceeding 50%, comparable to or greater than those of tricyclic antidepressants [49]. In previous studies, phenelzine demonstrated *K*i values of 146.0 ± 0.009 nM and 110.0 ± 0.005 nM against MAO‐A and MAO‐B, respectively, with mixed and irreversible inhibition [50]. Moreover, *IC*_50_ values of 0.221 ± 0.009 µM for MAO‐A and 0.131 ± 0.023 µM for MAO‐B further support its selection as a reference inhibitor [51]. For MAO‐A, Phenol, 2‐(1,1‐dimethylethyl)‐4‐(1,1,3,3‐tetramethylbutyl) (CID 605573) exhibited the most favorable binding affinity, with a docking score of −9.208 kcal/mol, indicating a strong predicted interaction with the enzyme. Although no hydrogen bonds were observed, the ligand formed extensive hydrophobic interactions with key active site residues, including TYR69, ILE180, ILE335, LEU337, MET350, PHE352, TYR407, and TYR444, which likely contribute significantly to its stability (Figure 5a). In comparison, 4‐methylbenzaldehyde (CID‐7725) showed a moderate binding score of −7.41 kcal/mol and formed hydrogen bonds with ALA68 and TYR69, along with hydrophobic interaction at TYR407, suggesting a balanced but weaker interaction profile (Figure 5b). Tetradecyl chloroacetate (CID‐519540) demonstrated a binding score of −6.901 kcal/mol and interacted predominantly through hydrophobic contacts with multiple residues such as TYR69, LEU97, PHE208, CYS323, ILE325, ILE335, LEU337, PHE352, TYR407, and TYR444, but lacked hydrogen bonding, which may limit its binding strength (Figure 5c). The control ligand, phenelzine (CID‐3675), showed the lowest binding affinity (−6.196 kcal/mol) among the tested compounds, despite forming several hydrogen bonds with residues ILE180, ASN181, ILE207, GLN215, and TYR444, and a hydrophobic interaction with PHE352, indicating comparatively weaker overall stability (Figure 5d).

For MAO‐B, methyl 2‐hydroxy‐octadeca‐9,12,15‐trienoate (CID‐91697556) displayed the highest binding affinity with a score of −10.478 kcal/mol, suggesting a very strong interaction with the enzyme. This compound formed hydrogen bonds with LEU171 and TYR435, along with extensive hydrophobic interactions involving residues such as TYR60, PHE103, PRO104, TRP119, LEU164, PHE168, CYS172, ILE198, ILE199, ILE316, and PHE343, indicating a highly stable and well‐accommodated binding within the active site (Figure 6a). Tetradecyl chloroacetate (CID‐519540) also showed strong binding (−8.978 kcal/mol), forming hydrogen bonds with TYR398 and TYR435 and hydrophobic interactions with key residues including PHE103, TRP119, LEU167, PHE168, LEU171, CYS172, ILE199, and ILE316 (Figure 6b). Similarly, 1‐Benzenesulfonamide, N1‐(2‐hydroxycyclohexyl)‐4‐methyl (CID‐575170) demonstrated a comparable binding score (−8.962 kcal/mol), with hydrogen bonding involving PRO102 and PRO104 and hydrophobic contacts with TRP119, LEU171, CYS172, ILE199, and TYR326 (Figure 6c). The control, phenelzine (CID‐3675), again showed the weakest interaction with a binding score of −6.437 kcal/mol, forming only one hydrogen bond with THR195 and limited hydrophobic interactions with PHE103, PRO104, LEU164, and ILE199 (Figure 6d). Altogether, the docking findings suggest that these compounds possess stronger binding affinity and more favorable interaction profiles compared to the standard inhibitor, highlighting their potential as promising MAO‐A and MAO‐B inhibitors for further investigation in PD therapy.

#### 3.4.3 ADMET Profiling

ADMET profiling is an important preliminary step in evaluating the pharmacokinetic and safety characteristics of potential drug candidates [52]. In this study, the Lipinski Rule of Five was applied to assess the drug‐likeness of compounds selected against MAO‐A and MAO‐B and to identify promising neuropharmacological leads. The predicted ADMET parameters are presented in Table 2. All selected compounds and reference drugs had molecular weights below 500 g/mol and exhibited no more than one Lipinski rule violation, with acceptable predicted bioavailability. None of the selected compounds showed predicted Ames toxicity, whereas the reference drug CID‐3675 was Ames‐positive. The compounds also displayed lower topological polar surface areas than the reference drugs and were predicted to be non‐hepatotoxic, suggesting a favorable preliminary safety profile regarding hepatic toxicity [53]. The predicted maximum tolerated human doses ranged from 0.025 to 1.146 log mg/kg/day, while rat LD_50_ values ranged from 1.601 to 2.868. Additional molecular descriptors, including rotatable bonds and hydrogen‐bond donor/acceptor counts, are summarized in Table 2.

**TABLE 2:** The physiochemical and pharmacological profile of the selected ligand molecules.

Physicochemical										Pharmacological					
Compound CID	MW	HA	HD	NRB	MoR	SA	NLV	DL	IA	BBB	TC	AT	LD_50_	HT	MDT
CID‐3675 (Control)	136.19	2	2	3	41.72	61.021	0	Yes	High	Yes	0.394	Yes	2.868	No	1.146
CID‐605573	262.43	1	1	4	85.97	118.61	1	Yes	High	Yes	0.58	No	2.36	No	0.517
CID‐7725	120.15	1	0	1	36.80	54.32	0	Yes	High	Yes	0.245	No	1.79	No	0.908
CID‐519540	290.87	2	0	15	85.11	123.79	1	Yes	High	Yes	0.825	No	2.12	No	0.195
CID‐91697556	308.46	3	1	14	94.47	135.309	0	Yes	High	Yes	2.149	No	1.601	No	−0.025
CID‐575170	269.36	4	2	3	70.39	108.085	0	Yes	High	Yes	1.388	No	2.234	No	0.534

Abbreviations: *ADMET: absorption, distribution, metabolism, excretion, and toxicity; MW: molecular weight; HA: No. of hydrogen bond acceptor; HD: No. of hydrogen bond donor; NRB: No. of rotatable bonds; MoR: molar refractivity; SA: surface area; NLV: No. of Lipinski's rule violations; DL: drug‐likeness; IA: intestinal absorption; BBB: blood–brain barrier; TC: total clearance, log mL/(min/kg); AT: AMES toxicity; LD_50_: oral rat acute toxicity, mg/kg; HT: hepatotoxicity; MTD: maximum tolerated dose for a human, log mg/(kg/day).

#### 3.4.4 Ligands Activity Analysis via PASS Prediction

The potential biological activities of the selected compounds were predicted using the PASS server based on their calculated Pa and Pi values, as summarized in Table 3. CID‐519540 demonstrated strong antiparkinsonian potential against tremor (Pa = 0.441, Pi = 0.008) and rigidity (Pa = 0.422, Pi = 0.016), along with diabetic neuropathy treatment (Pa = 0.330, Pi = 0.149). CID‐7725 exhibited antiparkinsonian rigidity relief (Pa = 0.332, Pi = 0.042) and tremor relief (Pa = 0.208, Pi = 0.069) and was uniquely predicted as a neurotrophic factor enhancer (Pa = 0.341, Pi = 0.009), along with diabetic neuropathy treatment (Pa = 0.334, Pi = 0.141). CID‐605573 showed strong activity for anti‐neurogenic pain (Pa = 0.456, Pi = 0.014) and acute neurologic disorders treatment (Pa = 0.450, Pi = 0.111), with weaker antiparkinsonian tremor relief (Pa = 0.151, Pi = 0.105). CID‐575170 displayed moderate activity for diabetic neuropathy treatment (Pa = 0.354, Pi = 0.103) and antiparkinsonian rigidity relief (Pa = 0.217, Pi = 0.118). CID‐91697556 demonstrated activity for acute neurologic disorders treatment (Pa = 0.500, Pi = 0.082) and diabetic neuropathy treatment (Pa = 0.305, Pi = 0.204). PASS analysis predicted that all selected compounds exhibit potential neurological and antiparkinsonian activities.

**TABLE 3:** PASS prediction of biological activities of selected ligands.

Compound CID	Probable activity (Pa)	Probable inactivity (Pi)	Activity
CID‐519540	0.441	0.008	Antiparkinsonian, tremor relieving
	0.422	0.016	Antiparkinsonian, rigidity relieving
	0.33	0.149	Diabetic neuropathy treatment
CID‐7725	0.341	0.009	Neurotrophic factor enhancer
	0.334	0.141	Diabetic neuropathy treatment
	0.332	0.042	Antiparkinsonian, rigidity relieving
	0.208	0.069	Antiparkinsonian, tremor relieving
CID‐605573	0.456	0.014	Anti‐neurogenic pain
	0.45	0.111	Acute neurologic disorders treatment
	0.151	0.105	Antiparkinsonian, tremor relieving
CID‐575170	0.354	0.103	Diabetic neuropathy treatment
	0.217	0.118	Antiparkinsonian, rigidity relieving
CID‐91697556	0.5	0.082	Acute neurologic disorders treatment
	0.305	0.204	Diabetic neuropathy treatment

#### 3.4.5 Molecular Dynamics (MD) Simulation

A 100 ns MD simulation was performed to assess the stability and conformational dynamics of selected MAO‐A and MAO‐B protein‐ligand complexes under physiologically relevant conditions. Complex stability and interactions were evaluated using RMSD, RMSF, SASA, PSA, MolSA, and intermolecular hydrogen‐bond analyses.

#### 3.4.6 Root Mean Square Deviation (RMSD) Analysis

RMSD is a commonly used metric for assessing the structural stability and conformational dynamics of protein‐ligand complexes by quantifying atomic deviations from the initial structure throughout the simulation [54]. The RMSD trajectories of MAO‐A complexes with CID‐7725, CID‐519540, CID‐605573, and CID‐3675 exhibited varying degrees of structural stability (Figure 7a). Among these, the complexes with CID‐7725 and CID‐519540 maintained relatively stable conformations, with fluctuations largely confined within the acceptable range of 2.1 Å–4.9 Å, indicating stable binding and minimal conformational drift throughout the simulation. In contrast, the complex with CID‐605573 showed comparatively higher fluctuations, with RMSD values ranging from 2.9 Å to 7.08 Å, suggesting increased structural flexibility and possible instability within the binding pocket. The control compound, CID‐3675, displayed moderate deviations, indicating a relatively stable but less optimal interaction compared to the better‐performing ligands.

**FIGURE 7:**
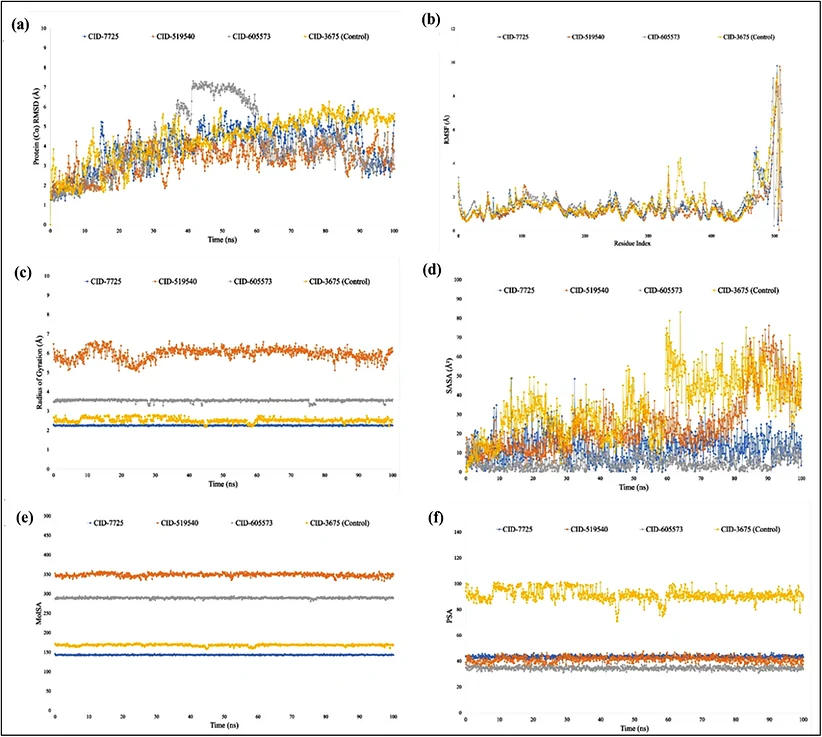
MD simulation analysis of selected compounds, including CID‐7725, CID‐519540, CID‐605573, and the control compound CID‐3675, in complex with MAO‐A; the trajectories are represented by blue, orange, grey, and yellow, respectively. The analysis includes (a) RMSD, (b) RMSF, (c) *R*_g_, (d) SASA, (e) MolSA, and (f) PSA.

Conversely, all MAO‐B complexes demonstrated consistent RMSD behavior, with values remaining within approximately 1.1 Å to 3.2 Å (Figure 8a). The narrow range of deviation observed across these systems indicates that the ligand–protein assemblies retained their structural integrity and exhibited stable binding interactions throughout the simulation period. This stability may be attributed to favorable interactions within the active site, leading to reduced conformational fluctuations. Additionally, the comparatively lower RMSD values for MAO‐B complexes suggest a more rigid and well‐accommodated binding environment than that observed for MAO‐A complexes. The RMSD results indicate that most ligand‐protein complexes remained structurally stable over time. Notably, MAO‐B complexes exhibited greater conformational stability than MAO‐A complexes, highlighting their potential for stronger and more consistent binding interactions under simulated physiological conditions.

**FIGURE 8:**
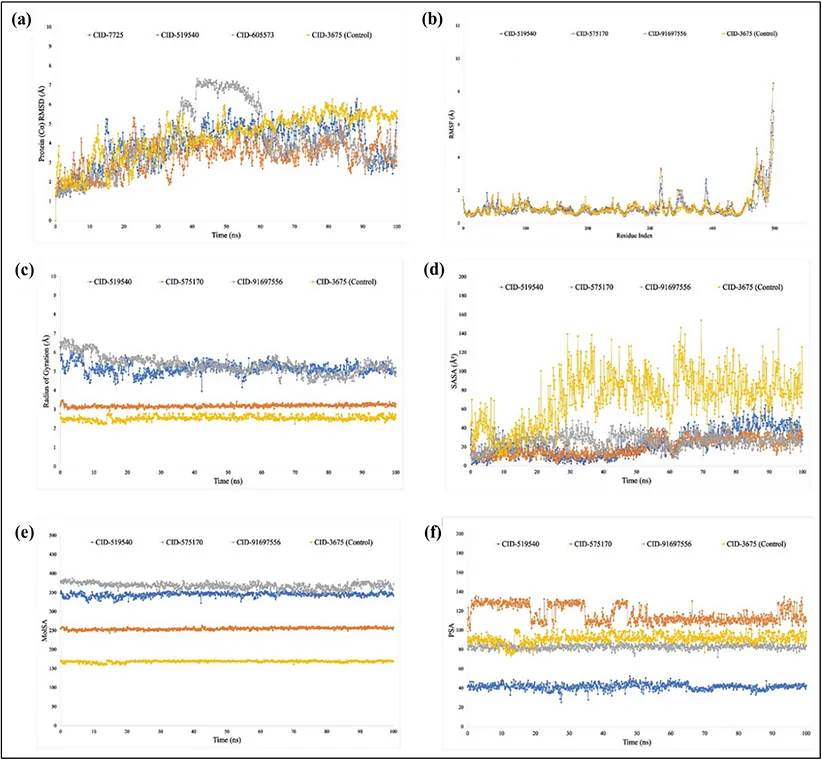
MD simulation analysis of selected compounds, including CID‐519540, CID‐575170, CID‐91697556, and the control compound CID‐3675, in complex with MAO‐B; the trajectories are represented by blue, orange, grey, and yellow, respectively. The analysis includes (a) RMSD, (b) RMSF, (c) *R*_g_, (d) SASA, (e) MolSA, and (f) PSA.

#### 3.4.7 Root Mean Square Fluctuation (RMSF) Analysis

Root mean square fluctuation (RMSF) is a widely applied measure for evaluating residue‐level flexibility by determining the average positional variation of individual amino acids relative to their reference positions throughout the simulation [55]. This parameter provides detailed insight into local conformational changes within protein–ligand complexes and enables the identification of flexible and stable regions of the protein upon ligand binding.

The RMSF profiles of MAO‐A complexes revealed noticeable fluctuations across several regions of the protein backbone (Figure 7b). Residues 463–512 exhibited comparatively higher flexibility across all ligand‐bound systems, suggesting that this region may play a role in dynamic conformational adjustments during ligand interaction. Despite these localized fluctuations, most residues maintained relatively low RMSF values, indicating overall structural stability of the protein during the simulation. Among the evaluated ligands, the complex with CID‐605573 showed comparatively greater residue‐level fluctuations, with a pronounced peak around residue 317, indicating increased local flexibility and possible conformational rearrangements at this position. This behavior may reflect weaker or less stable interactions within specific regions of the binding site.

In contrast, all MAO‐B complexes displayed relatively minor fluctuations overall, with limited variations primarily observed within the residue range of 310–362 (Figure 8b). The lower RMSF values across most residues suggest a more rigid and stable protein structure upon ligand binding. This reduced flexibility may be indicative of stronger ligand accommodation within the active site, leading to fewer structural perturbations during the simulation. Collectively, the RMSF results indicate that MAO‐B complexes exhibit greater structural stability and reduced residue‐level mobility compared to MAO‐A complexes, further supporting their favorable binding behavior.

#### 3.4.8 Radius of Gyration (*R*_g_) Analysis

Radius of gyration (*R*_g_) is a useful structural parameter for assessing the compactness and conformational stability of protein–ligand complexes by measuring the spatial distribution of atoms around the system's center of mass during MD simulations. Changes in *R*_g_ provide insights into structural integrity, folding behavior, and overall conformational stability of the macromolecular complex.

The *R*_g_ values of the MAO‐A and MAO‐B complexes fluctuated approximately between 2.3 and 6.3 Å throughout the simulations (Figures 7c and 8c), indicating overall maintenance of structural compactness. The limited variation in Rg suggests that ligand binding did not cause substantial global unfolding or destabilization of the protein structures. Overall, both proteins retained their compact conformations, indicating stable folding behavior in the presence of the tested ligands. Minor variations observed in Rg values may be attributed to local conformational adjustments required to accommodate ligand interactions within the active site. The *R*_g_ analysis supports the notion that the protein‐ligand complexes remain structurally stable during the simulation, with no substantial loss of compactness, further corroborating the stability observed in RMSD and RMSF analyses.

#### 3.4.9 Solvent‐Accessible Surface Area (SASA)

Solvent‐accessible surface area (SASA) analysis is an important parameter for characterizing the structural and functional properties of biomolecular systems by measuring the surface area exposed to the surrounding solvent. For protein–ligand complexes, SASA helps assess the accessibility of binding‐site residues and their potential involvement in ligand interactions [56].

The SASA profiles of MAO‐A and MAO‐B complexes revealed notable interactions between amino acid residues and the bound ligands. The observed SASA values ranged from approximately 1 to 45 Å^2^ (Figures 7d and 8d), indicating moderate to high surface exposure of key residues involved in ligand binding. These fluctuations reflect the conformational adaptability of the protein structures upon ligand interaction, which may influence binding stability and overall MD. Notably, slight deviations in SASA patterns were observed in the MAO‐A complex with CID‐519540 as well as the control system, suggesting differences in residue exposure and interaction behavior compared to the other complexes. These variations may be associated with differences in ligand accommodation within the binding pocket and the degree of solvent exposure of interacting residues. The SASA analysis supports the structural stability of the protein–ligand systems and highlights the role of solvent accessibility in modulating interaction dynamics and ligand binding behavior.

#### 3.4.10 Molecular Surface Area (MolSA and Polar Surface Area (PSA) Analysis

Molecular surface area (MolSA) analysis was used to characterize the van der Waals surface and molecular packing of the protein–ligand complexes. MolSA was calculated using a 1.4 Å probe radius to estimate the interaction surface. The MAO‐A and MAO‐B complexes showed relatively consistent MolSA profiles throughout the simulations (Figures 7e and 8e), suggesting stable molecular packing and favorable surface complementarity between the selected ligands and their respective target proteins.

Polar surface area (PSA) analysis, which is primarily influenced by electronegative atoms such as nitrogen and oxygen, provides important information regarding the polarity and interaction potential of the complexes. The evaluated systems showed appreciable PSA values (Figures 7f and 8f), suggesting that polar functional groups contribute to the stabilization of protein–ligand interactions through hydrogen bonding and electrostatic forces. The MolSA and PSA profiles indicate favorable structural compatibility and interaction potential of the selected compounds with MAO‐A and MAO‐B, supporting the binding patterns observed in the molecular docking and MD simulations.

#### 3.4.11 Intramolecular Bonds Analysis

Intramolecular interaction analysis plays a crucial role in understanding the stability and binding behavior of protein–ligand complexes. Among these interactions, hydrogen bonds and hydrophobic contacts are the primary forces contributing to ligand binding and stabilization within the active site. The intermolecular hydrogen‐bond and hydrophobic interaction profiles observed throughout the 100 ns MD simulations are presented in Figures 9 and 10 for the MAO‐A and MAO‐B complexes, respectively. The analyzed complexes exhibited distinct interaction patterns, reflecting variations in ligand orientation and binding modes within the protein structures. Notably, the CID‐519540‐MAO‐B complex demonstrated the most prominent interaction profile, characterized by a higher number of stabilizing interactions, including hydrogen bonds, hydrophobic contacts, ionic interactions, and water‐mediated bridges (Figure 10a). This extensive interaction network suggests enhanced binding stability and favorable accommodation of the ligand within the MAO‐B binding pocket. In contrast, the CID‐519540‐MAO‐A, CID‐605573‐MAO‐A, and CID‐575170‐MAO‐B complexes exhibited comparatively fewer interactions, with a reduced number of interacting residues and less uniform distribution of binding contacts (Figures 9b,c, and 10b). These differences may be attributed to variations in ligand conformational flexibility and binding orientation within the respective active sites, leading to less optimal interaction networks. The interaction analysis highlights the importance of multiple non‐covalent interactions in stabilizing protein–ligand complexes and supports the observed differences in binding behavior among the studied systems.

**FIGURE 9:**
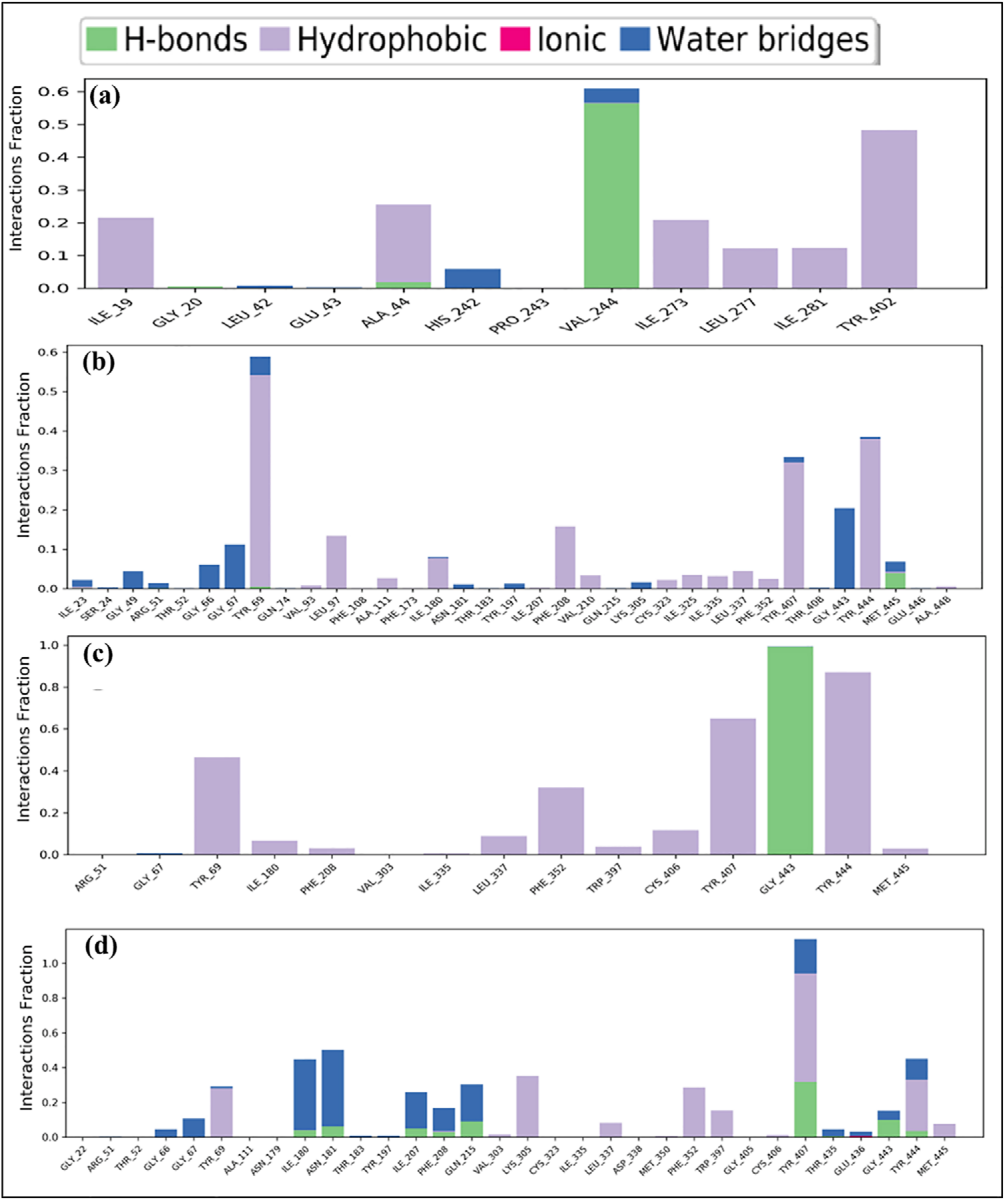
Protein–ligand interactions during 100 ns MD simulation of MAO‐A (PDB ID: 2Z5X) with selected ligands. Stacked bar charts show the total number of intermolecular hydrogen bonds and hydrophobic interactions formed between MAO‐A and (a) CID‐7725, (b) CID‐519540, (c) CID‐605573, and (d) CID‐3675 (phenelzine control).

**FIGURE 10:**
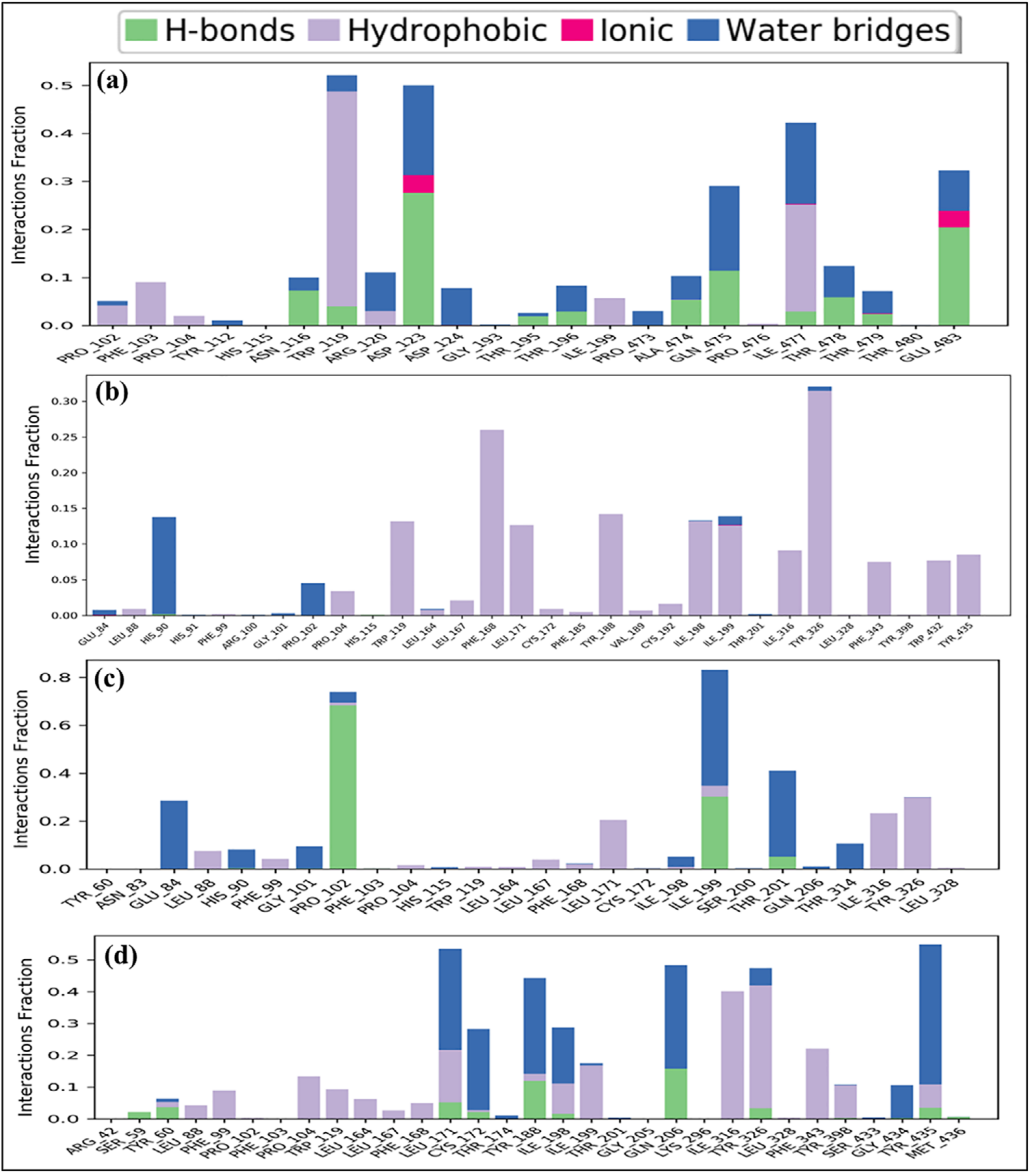
Protein–ligand interactions during 100 ns MD simulation of MAO‐B (PDB ID: 4A79) with selected ligands. The stacked bar charts show the total number of intermolecular hydrogen bonds and hydrophobic interactions formed between MAO‐B and (a) CID‐519540, (b) CID‐575170, (c) CID‐91697556, and (d) CID‐3675 (phenelzine control).

## 4 Discussion

The rising global burden of PD has encouraged the development of natural multi‐target therapeutic agents that may provide safer and more effective alternatives to conventional synthetic drugs [1, 2, 57].

The present study suggests *L. sinense* as a potential neuropharmacological candidate. The extract showed moderate DPPH radical scavenging (IC_50_ = 73.56 ± 2.18 µg/mL) versus ascorbic acid (7.29 ± 2.86 µg/mL) (Figure 1), with TPC (151.6 mg GAE/g) and TFC (67.25 mg QE/g) suggesting phenolic compounds as primary antioxidants. These results corroborate [22], who isolated antioxidative glycosides from *L. sinense*, and [23], who reported flavonoids and phenylethanoic glycosides with antioxidant/anti‐inflammatory activities in *Ligustrum* species. Since oxidative stress drives dopaminergic degeneration in PD via ROS‐mediated damage [8, 9], *L. sinense's* antioxidant properties may enhance its neuroprotective potential.

The in vivo evaluation revealed that *L. sinense* did not produce significant sedative effects at 200–400 mg/kg, unlike diazepam, which significantly reduced locomotor activity (Figure 2a,b). This absence of sedative effects aligns with multiple phytotherapeutics that dissociate anxiolysis from sedation [58, 59]. Such dissociation between anxiolysis and sedation is therapeutically advantageous, particularly for PD patients, where excessive sedation would exacerbate motor dysfunction and fall risk [3, 5]. The lack of sedation in *L. sinense* may be attributed to its specific phytochemical composition, which likely lacks potent GABAergic agonists, the primary mediators of benzodiazepine‐induced sedation, with anxiolytic effects mediated instead through MAO inhibition or serotonergic modulation [60, 61]. Furthermore, oral administration of crude extract may result in lower bioavailability of sedative constituents compared to intraperitoneal diazepam [28], and the complex phyto‐complex may contain opposing compounds that counteract sedation [62].

Despite the absence of sedative effects, *L. sinense* exhibited a distinct anxiolytic‐like profile. The extract significantly increased open arm time in the EPM at 200 mg/kg (*p< 0.05) and 400 mg/kg (**p< 0.01) (Figure 3a) and increased head‐dipping in the HBT at both doses (*p< 0.05 and **p < 0.01) (Figure 3c). These findings indicate genuine anxiolytic effects comparable to those reported for *Passiflora incarnata*  [59] and are consistent with the anti‐inflammatory properties documented in *Ligustrum* species [21]. The observed separation of anxiolytic efficacy from sedative liability supports the therapeutic potential of *L. sinense* for anxiety management in PD, a critical non‐motor symptom without compromising motor function [63]. This anxiolytic effect is particularly relevant to PD, as anxiety disorders affect up to approximately 31% of PD patients and are more closely linked to degeneration of serotonergic and noradrenergic, rather than purely dopaminergic, pathways [64, 65]. Clinical evidence for a direct anxiolytic benefit of selective MAO‐B inhibitors in PD has been mixed, whereas MAO‐A inhibition is more directly implicated in serotonergic and noradrenergic tone; this mechanistic distinction strengthens the rationale for the dual MAO‐A/MAO‐B inhibitory profile of *L. sinense* phytocompounds observed here as a targeted, rather than incidental, therapeutic mechanism. MAO‐A inhibition is well established to elevate synaptic serotonin and noradrenaline levels, the basis for its recognized antidepressant and anxiolytic pharmacology [66].

Building on these in vivo findings, in silico docking analysis provides mechanistic insight into MAO inhibition. Both MAO isoforms metabolize monoamine neurotransmitters relevant to PD: MAO‐B is the predominant isoform in the substantia nigra and preferentially deaminates dopamine, making it the primary therapeutic target for motor symptoms [15, 18]. Accordingly, all FDA‐approved MAO inhibitors for PD selectively target MAO‐B (e.g., selegiline, rasagiline) [4].

Among 20 phytocompounds analyzed, methyl 2‐hydroxy‐octadeca‐9,12,15‐trienoate (CID‐91697556) exhibited the strongest MAO‐B binding (‐10.478 kcal/mol), (Table 1) with hydrogen bonds to LEU171 and TYR435 interactions consistent with the binding mode of glitazone class MAO‐B inhibitors [38]. This binding affinity compares favorably to quercetin, a well‐characterized natural MAO‐B inhibitor [67]. While MAO‐B is the primary focus for PD motor symptoms, MAO‐A inhibition may contribute to managing non‐motor features. MAO‐A preferentially metabolizes serotonin and norepinephrine, and its inhibition is associated with anxiolytic effects [18]. Our behavioral data showing significant anxiolytic activity in the EPM and HBT (Figure 3a–c) may partly reflect MAO‐A modulation. Notably, Tetradecyl chloroacetate (CID‐519540) demonstrated dual MAO‐A/MAO‐B inhibition (−6.901 and −8.978 kcal/mol, respectively), an attribute that could address both motor and non‐motor symptom domains an emerging therapeutic strategy in PD [63, 68].

ADMET profiling confirmed favorable drug‐like properties for lead compounds, including blood–brain barrier permeability and absence of hepatotoxicity or mutagenicity (Table 2). This favorable in silico safety profile is consistent with the long traditional use of *L. sinense* leaves as an ingredient of Ku‐Ding‐Cha, a bitter herbal tea consumed in China without reported serious adverse effects [24], although formal clinical toxicology data for the extract itself remain to be established. In line with this favorable profile, brine shrimp lethality testing of the extract previously yielded an LC_50_ of 430.94 ± 0.54 µg/mL, reflecting minimal acute toxicity [35]. These properties are critical for CNS‐active drugs targeting PD [53, 69]. PASS prediction further supported the neurotherapeutic potential of these compounds (Table 3). CID‐519540 demonstrated strong antiparkinsonian activity against tremor (Pa = 0.441) and rigidity (Pa = 0.422). CID‐91697556 showed the highest probability for acute neurologic disorders treatment (Pa = 0.500) [11]. The stability of MAO‐B binding was rigorously validated through MD simulations: CID‐91697556 exhibited exceptional complex stability over 100 ns (RMSD: 1.1–3.2 Å) (Figure 6a and 7a), with narrow *R*_g_ fluctuations (2.3–6.3 Å) (Figure 6c and 7c), confirming maintained structural compactness without protein unfolding. In summary, while *L. sinense* phytocompounds interact with both MAO isoforms, the exceptionally stable MAO‐B binding (RMSD: 1.1–3.2 Å; score: −10.478 kcal/mol) positions CID‐91697556 as a promising lead for PD drug development, with CID‐519540 offering additional dual‐inhibition potential.

This study integrates in vivo behavioral pharmacology with in silico molecular modeling to demonstrate that *L. sinense* possesses a multi‐target neuropharmacological profile relevant to PD. The extract exhibits moderate antioxidant activity, dose‐dependent anxiolytic effects without sedation, and phytocompounds with high‐affinity binding to MAO‐B validated through stable MD simulations. The identification of CID‐91697556 as a stable MAO‐B inhibitor and CID‐519540 as a dual MAO‐A/B inhibitor positions *L. sinense* as a promising natural source for developing PD therapeutics that could address both motor symptoms (via MAO‐B inhibition) and non‐motor anxiety (via MAO‐A/serotonergic modulation), without the sedative liabilities of conventional anxiolytics.

## 5 Future Research and Limitations

Several limitations should be acknowledged. The in vivo study employed healthy animals rather than validated PD models and used only two acute doses (200 and 400 mg/kg), limiting dose‐response and translational interpretation. Behavioral findings were not supported by biochemical, molecular, or direct MAO‐A/MAO‐B inhibition assays, and assessments were not performed under blinded conditions, potentially introducing observer bias. Although the sample size was based on the resource equation approach, an a priori power analysis was not conducted, and assumptions underlying parametric ANOVA were not formally verified. The use of a crude, non‐standardized extract and a single DPPH assay also limits compound‐specific mechanistic interpretation. Furthermore, molecular docking results were not supported by MM/GBSA or MM/PBSA binding free‐energy calculations, which could have provided additional quantitative validation of ligand–protein interactions. Future studies should incorporate standardized extracts, complementary antioxidant assays, validated PD models, broader dose‐response studies, direct MAO‐A/MAO‐B inhibition assays, biochemical and molecular analyses, blinded behavioral assessments, rigorous statistical validation, and comprehensive in vivo toxicological evaluation.

## 6 Conclusion

The present study demonstrated that *L. sinense* leaf extract possesses moderate antioxidant and notable anxiolytic‐like activities without inducing significant sedative effects across multiple behavioral models. Furthermore, in silico molecular docking, ADMET profiling, PASS prediction, and MD simulations revealed favorable interactions between several identified phytocompounds and MAO‐A/MAO‐B enzymes, supporting their potential role as MAO inhibitors. Among the compounds, CID‐91697556 was identified as a stable MAO‐B inhibitor, while CID‐519540 exhibited dual inhibitory potential against both MAO‐A and MAO‐B. The antioxidant activity observed in the DPPH assay further suggests a possible role in reducing oxidative stress associated with neurodegenerative disorders, including Parkinson's disease (PD). Overall, these findings indicate that *L. sinense* may serve as a promising natural source of bioactive compounds with neuropharmacological potential relevant to PD. Nevertheless, further in vitro, mechanistic, pharmacokinetic, and disease‐specific studies are necessary to validate these findings and clarify the therapeutic applicability of *L. sinense*. Clinically, these findings suggest that *L. sinense* extract, or its lead phytocompounds such as CID‐91697556 and CID‐519540, could eventually be explored as adjunct agents capable of addressing both motor symptoms, through MAO‐B inhibition, and the anxiety that affects a substantial proportion of PD patients, through MAO‐A‐related mechanisms, potentially without the sedative burden associated with conventional anxiolytics such as benzodiazepines. Realizing this clinical potential, however, will depend on the mechanistic, pharmacokinetic, and disease‐model validation outlined above.

## Author Contributions

**Sowmitro Das**: conceptualization, methodology, investigation, formal analysis, writing – original draft, and writing – review and editing. **S. M. Istiaque Hamim**: conceptualization, methodology, investigation, formal analysis, data curation, visualization, writing – original draft, and writing – review and editing. **Md Nazmul Hasan Zilani**: conceptualization, validation, and writing – review and editing. **Kazi Sneha Chhando**: methodology, formal analysis, data curation, visualization, and writing – review and editing. **Puza Das, Md Ridoy Hossain, Md. Imtiaz, and Jebin Tasmin**: formal analysis, and writing – review and editing. **Roney Miah**: supervision, resources, data curation, validation, and writing – review and editing; **Md. Nazmul Hasan**: supervision, resources, acquired funding, project administration, and writing – review and editing. All authors have approved the final version of the manuscript.

## Funding

This study was partially supported by Ministry of Posts, Telecommunications and Information Technology, Department of Information and Communication Technology, Bangladesh (ID: 22‐40); Ministry of Science and Technology, Bangladesh (Grant: MoST/2021–22/BS‐277).

## Ethics Statement

The experimental procedures conducted in vivo adhered to the ethical standards approved by the Ethical Review Committee of the Faculty of Biological Science and Technology at Jashore University of Science and Technology [Ref: ERC/FBS/JUST/2022–114].

## Conflicts of Interest

The authors declare no conflicts of interest.

## Use of Generative AI and AI‐Assisted Technologies in the Writing Process

The authors used ChatGPT (GPT‐5.5 Instant, OpenAI) and Grammarly Free (Basic) as AI‐based language editing tools to improve the clarity, grammar, and readability of the manuscript. These tools were used solely for language editing and refinement. No AI tools were used for data analysis, interpretation of results, or generation of scientific content. All authors critically reviewed and approved the final version of the manuscript.

## Supporting information




**Supporting File**: cbdv71799‐sup‐0001‐SuppMat.xlsx.

## Data Availability

The data that support the findings of this study are available from the corresponding author upon reasonable request.
